# Adsorption of antimicrobial peptide onto chitosan-coated iron oxide nanoparticles fosters oxidative stress triggering bacterial cell death†

**DOI:** 10.1039/d3ra04070d

**Published:** 2023-08-25

**Authors:** Lipsa Leena Panigrahi, Shashank Shekhar, Banishree Sahoo, Manoranjan Arakha

**Affiliations:** a Center for Biotechnology, Siksha ‘O’ Anusandhan (Deemed to be University) Bhubaneswar 751003 Odisha India marakha@soa.ac.in; b Indian Institute of Technology Hyderabad India

## Abstract

In the prevailing environmental status quo, bacterial resistance has made antibiotics and antimicrobial peptides (AMPs) ineffective, imparting a serious threat and putting a much greater financial burden on the biomedical and food industries. For this reason, the present study investigates the potential of iron oxide nanoparticles (IONPs) coated with chitosan (CS-IONP) as a platform for augmenting the antimicrobial activity of antimicrobial peptides like nisin. Hence, the nisin is allowed to be adsorbed onto chitosan-coated IONPs to formulate nisin-loaded CS-IONP nanoconjugates. The nanoconjugates were characterized by various optical techniques, such as XRD, FTIR, SEM, zeta and DLS. Remarkably, lower concentrations of N-CS-IONP nanoconjugate exhibited significant and broad-spectrum antibacterial potency compared to bare IONPs and nisin against both Gram-positive and Gram-negative bacteria. Biofilm production was also found to be drastically reduced in the presence of nanoconjugates. Further investigation established a relationship between an increase in antibacterial activity and the enhanced generation of reactive oxygen species (ROS). Oxidative stress exhibited due to enhanced ROS generation is a conclusive reason for the rupturing of bacterial membranes and leakage of cytoplasmic contents, eventually leading to the death of the bacteria. Thus, the current study emphasizes the formulation of a novel antimicrobial agent which exploits magnetic nanoparticles modulated with chitosan for enhanced remediation of resistant bacteria due to oxidative stress imparted by the nanoconjugates upon interaction with the bacteria, leading to cell death.

## Abstract 2

A facile approach towards the formulation of antimicrobial peptide adsorbed magnetic nano-conjugates with oxidative stress mediated bacterial cell death.

## Graphical abstract

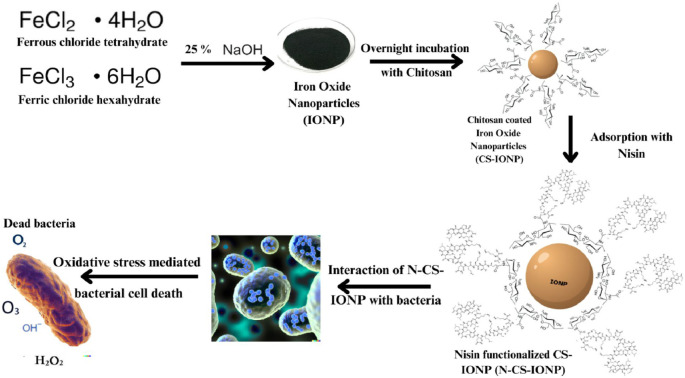

## 1. Introduction

Multidrug-resistant microorganisms and heightened pathogenicity of existing microorganisms is a global challenge to be mitigated by the current biomedical sector. Microorganisms exhibit remarkable resilience and adaptability and possess the ability to undergo genetic and phenotypic changes that enable them to survive and thrive in different environments, including in the presence of different antimicrobial agents.^1^ The same microbes can acquire multiple-drug resistant genes from various organisms, resulting in the creation of multidrug resistant (MDR) “superbugs”.^2^ According to recent statistics, antibiotic-resistant bacteria cause about 2 million episodes of serious illness, including 23 000 deaths each year in the US alone.^2^ In most cases, MDR infections necessitate prolonged antibiotic therapy and high health-care costs of up to approx. $55 billion each year.^2^ Hence, the formulation of antimicrobial agents/strategies for the elimination of multidrug-resistant microorganism has drawn the attention of current researchers. In this context, magnetic nanoparticles (MNPs) are a class of metal nanoparticle that can be manipulated using an alternating current or magnetic field, making them useful for various biomedical applications.^3^ Iron oxide nanoparticles (IONPs) are currently popular due to their biocompatibility and ease of synthesis; hence, they are rapidly being put to use for magnetic cell labelling, tracking and separation, as catalysts and biomarkers, for drug delivery and as antimicrobial agents after surface functionalization.^3^ Surface coating with diverse inorganic and organic components is required for the utilization of IONPs in different biological applications.^4^ Furthermore, owing to their versatility, IONPs are currently being utilized in targeted drug delivery systems. IONPs can be directed to certain cells or tissues in the body by functionalizing the surface of the IONP with specific ligands.^4^ This focused strategy allows for more precise drug distribution, reducing adverse effects and maximising therapeutic advantages.^4^ Additionally, IONPs appear to be attractive candidates for hyperthermia therapies which are currently widely exploited for cancer treatment.^4^ For instance, superparamagnetic IONPs generate localized heat when subjected to alternating magnetic fields. They are employed to eliminate cancer cells while sparing healthy tissues, perhaps providing a less intrusive and more tailored therapeutic alternative. Magnetic resonance imaging (MRI) is a well-known biological use of IONPs. Coated IONPs can act as effective contrast agents, improving image resolution and providing significant information for diagnostic purposes due to their superparamagnetic properties and biocompatibility.^4^

In spite of their wide biomedical applications, the use of IONPs as an efficient antimicrobial agent is debatable. The primary reason is the size-dependent property of the nanoparticles, which increases their dissolution, which in turn allows the smaller particles to readily dissolve and release ions.^5^ This enhanced dissolution explains the toxicity of superparamagnetic iron oxide nanoparticles (SPIONs) towards various species of Gram-positive and Gram-negative bacteria by enhanced ROS generation and Fenton-reaction-based killing.^5^ However IONPs of larger size exhibit antimicrobial activity only at higher concentrations.^6^ This activity of IONP is constantly being modified by researchers by the attachment of suitable functionalities simply to change the interaction pattern at the bio–nano interface, thereby enhancing the chances of heightened antimicrobial activity. Additionally, IONPs can be easily coated with various materials to achieve stability.^7–9^ Another prime reason behind the popularity of IONPs in the biomedical field is that they are benign to the body, unlike other magnetic materials such as CaO nanoparticles which are toxic.^10^ Owing to their nano-dimensions, the encapsulated drugs/peptides/proteins are facilitated with a faster targeting and magnetic property, enabling smooth recovery *via* a strong external magnet.^11^ For example, studies have shown that iron oxide magnetic nanoparticles loaded with nisin and nystatin have antimicrobial properties against Gram-positive bacteria.^12,13^ This suggests that these functionalized nanoparticles have potential applications as antimicrobials in innovative and emerging technologies that utilize magnetic nanomaterials.^12^ Antimicrobial peptides (AMPs) have emerged as prospective treatments, exhibiting broad-spectrum efficacy and minimal acquisition of resistance due to their peculiar amphiphilic structure, with polycationic headgroups, enabling them to rupture microbial membranes. Nanomaterials are basically well suited to providing structural benefits to AMPs, therefore offering a viable answer for advanced antimicrobial therapy.

Within this framework, nisin, a cationic peptide consisting of 34 amino acid residues, known for its remarkable antimicrobial properties against Gram-positive bacteria and non-toxicity towards humans, is being used.^12^ There is great interest in using IONPs as a base material for the fabrication of a novel nanoconjugate. In the present study, we have synthesized IONPs using a co-precipitation method and the IONP surface has been functionalized using deacylated chitosan and thereby conjugated with an AMP like nisin. The resulting nanoconjugates have been well analysed for their proper adsorption, stability and size ratio by using various optical techniques like X-ray diffraction (XRD), Fourier transform infrared (FTIR), zeta, dynamic light scattering (DLS) and field emission scanning electron microscopy (FE-SEM). To optimise the nanoconjugates for various biomedical applications, their antibacterial and antibiofilm efficacies were evaluated against both Gram-positive and Gram-negative strains. Additionally, the underlying mechanism for the antimicrobial activity of the nanoconjugate was evaluated by ROS assay and leakage of cytoplasmic contents both qualitatively and quantitatively. The cytotoxicity analysis was carried out against a human embryonic kidney (HEK 293) cell line to evaluate the compatibility of the IONPs. This study represents the first attempt at developing such a nanoconjugate with enhanced efficacy with implications for various biomedical fields.

## 2. Experimental

In this study, reagent-grade chemicals, such as ferrous chloride tetrahydrate (FeCl_2_·4H_2_O), ferric chloride hexahydrate (FeCl_3_·6H_2_O), sodium hydroxide (NaOH), sodium phosphate dibasic heptahydrate, sodium phosphate monobasic monohydrate, and chitosan (deacetylated at a level of 90–95%), were obtained from Sigma-Aldrich. 7′-Dichlorodihydrofluorescein diacetate (DCFH-DA) dye used for the ROS study was procured from Cayman Chemicals, USA. Acetic acid (99.8%) was obtained from HiMedia Pvt. Ltd India. All experiments were conducted using deionized water. Bacterial cultures of *Micrococcus luteus* (MTCC 106), *Vibrio cholera* (MTCC 3906), *Escherichia coli* (MTCC-443), and *Staphylococcus epidermidis* (MTCC 435) were obtained from the Institute of Microbial Technology (IMTECH), Chandigarh, India.

### 2.1. Formulation of iron oxide nanoparticles (IONPs)

The IONPs were synthesized using the co-precipitation method, following the protocol outlined by Arakha *et al.*^14^ The co-precipitation involved the simultaneous precipitation of ferrous and ferric ion salts in an alkaline solution at ambient temperature. Specifically, Fe^2+^ (0.1 M) and Fe^3+^ (0.2 M) were dissolved in deionized water to form a solution. Briefly, for our experiment, 0.1 M ferrous chloride tetrahydrate (FeCl_2_·4H_2_O) and 0.2 M ferric chloride hexahydrate (FeCl_3_·6H_2_O) were added to 100 mL of deionized water. The solution was stirred thoroughly for proper mixing of the salts followed by heating at 60 °C under sealed conditions. 2.0 M NaOH was added to the mixture, resulting in a black-coloured solution. The pH of the solution was maintained at 11. The mixture is represented by the following reaction

1 2Fe^3+^ + Fe^2+^ + 8OH^−^ → Fe_3_O_4_ + 4H_2_O

The IONPs were separated by centrifuging them at 10 000 rpm for 10 minutes followed by washing. The acquired residues were then dried at 60 °C.

### 2.2. Functionalization of IONPs with chitosan (CS-IONPs)

The synthesized IONPs were functionalized with chitosan according to the following procedure: 80 mg of chitosan powder (CS) was dissolved in 1 M acetic acid solution to prepare CS solution, which was then added to a well-sonicated aqueous suspension of IONPs.^14^ The ratio of IONPs to CS was 5 : 2. The resulting mixture of CS-IONPs was stirred on a magnetic stirrer at room temperature for 18 hours, then separated through centrifugation at 10 000 rpm and dried at 70 °C for 2 hours.

### 2.3. Preparation of nisin-loaded CS-IONP nanoconjugates

To prepare the nisin-loaded CS-IONP (N-CS-IONP) conjugates, 1 mg mL^−1^ nisin was prepared in phosphate buffer (pH 7.4). The nisin solution was separately mixed with two different concentrations of CS-IONPs (2 mg mL^−1^ and 4 mg mL^−1^) and continuously stirred at 120 rpm at room temperature for 18 hours to facilitate nisin adsorption upon the CS-IONPs. The resulting nisin-chitosan-IONP (N-CS-IONP) nanoconjugates were then centrifuged and washed twice with PBS buffer to remove the unbound peptide and used for further analysis. The final suspensions were labelled N-CS-IONP (1 : 2) and N-CS-IONP (1 : 4).

### 2.4. Characterization of nisin-chitosan-IONP nanoconjugates

Initially, the crystallinity and different phases of the IONPs and CS-IONPs were studied with an X-ray diffractometer (Rigaku Miniflex 600, Japan) utilizing a powder X-ray diffraction pattern technique. Cu-Kα radiation with *λ* = 1.5406 Å was employed as the source of the monochromatic X-ray beam, and the data was obtained in the 2*θ* range of 10°–80° with a step size of 0.02° [2*θ*], and a scan rate of 3° per min. The samples were measured at a voltage of 40 kV and a current of 20 mA. The data obtained were further analysed utilizing PANAnalytical X'Pert HighScore software. The IR spectra were recorded on a PerkinElmer (US) FT-IR spectrophotometer in the range of 4000–400 cm^−1^. The zeta potential of the fabricated nanocomposite was measured with the help of a zeta potential analyzer (Zeecom Instrument). The dielectric constant for the experiment was 78.26. The cell thickness and width were 0.205 mm and 10.00 mm, respectively, with SL (front) of 0.043 mm, and SL (back) of 0.162 nm. The electrode distance was 3.60 cm. The voltage at which the zeta potential experiment was conducted was set at 110 V. The hydrodynamic sizes of the fabricated nanomaterials were measured by dynamic light scattering (DLS) using DLS instruments with a laser wavelength of 632.8 nm, and scattering angle of 90°, and the refractive index of the dispersant medium was 1.33. For the zeta potential analysis and DLS analysis, the nanoconjugates were uniformly dispersed in phosphate buffer (pH 7.4) and sonicated for 20 minutes before characterization. The morphological features of the formulated nanocomposites were analysed after gold coating for 3 min, at an accelerating voltage of 10 kV using FE-SEM (FE-SEM, Nova Nano SEM 450, FEI Company, Netherlands).

### 2.5. Effect of IONP, CS-IONP, and N-CS-IONP nanoconjugates on bacterial cell viability

#### 2.5.1. Growth kinetics assay

To assess the impact of IONP, CS-IONP, and N-CS-IONP nanoconjugates on bacterial cell viability, growth kinetics assay was conducted. To obtain bacterial culture for further experiments, a single colony from the nutrient agar plate was chosen and used for inoculation in 5 mL of nutrient broth medium. The culture was incubated at 37 °C with shaking at 120 rpm for 18 hours. The resulting bacterial culture was then adjusted to an optical density of 1.0 at 600 nm wavelength, which corresponds to a bacterial density of 10^8^ CFU mL^−1^. This suspension was then serially diluted using nutrient broth over a 2 log range, resulting in a bacterial density of 10^6^ CFU mL^−1^. To evaluate the antimicrobial activity of IONP, CS-IONP and N-CS-IONP nanoconjugates against microorganisms, a concentration-dependent experiment was initially designed. To be precise, different concentrations of IONPs and CS-IONPs (12.5 μg mL^−1^, 25 μg mL^−1^, 50 μg mL^−1^, 100 μg mL^−1^) and N-CS-IONPs (12.5 μL, 25 μL, 50 μL, 100 μL) were incubated with *S. epidermidis* and their growth kinetics were monitored at one-hour intervals using a Bio-Rad iMark plate reader. Additionally, the antimicrobial activity of the formulated N-CS-IONP nanoconjugates was evaluated against Gram-positive bacteria such as *S. epidermidis* and *S. aureus*, and Gram-negative bacteria such as *E. coli* by exposing them to 50 μL of different N-CS-IONP nanoconjugates.

#### 2.5.2. ROS detection assay

To assess the level of production of reactive oxygen species (ROS) following the interaction of N-CS-IONP nanoconjugate with bacteria, 2′,7′-dichlorodihydrofluorescein diacetate (DCFH-DA) was employed. Both Gram-positive and Gram-negative bacteria were used as test organisms. For the ROS quantification assay, *E. coli* and *S. epidermidis* bacterial cells were cultured in a 96-well plate and treated with 50 μL of N-CS-IONP (1 : 2) and N-CS-IONP (1 : 4) nanocomposites. After 3 hours of incubation, 5 μL of 10 mM of DCFH-DA stock solution was added. Reaction mixtures containing only IONP and only nisin were taken as negative controls and fluorescence emission was observed at 523 nm with excitation at 503 nm using a Synergy H1 microplate reader (BioTek, USA).

#### 2.5.3. Leakage of cytoplasmic contents

*E. coli* and *S. epidermidis* were grown in nutrient broth and left overnight to estimate the cytoplasmic leakage of nuclei acids and proteins upon interaction with nanoconjugates. The microbial culture was harvested by centrifuging it at 5000 rpm for 10 minutes. After washing, the pellets were resuspended in PBS buffer (pH 7.4). The bacterial cell count was adjusted to 10^5^ cells per mL. Cell suspensions were treated with N-CS-IONP (1 : 2) and N-CS-IONP (1 : 4) and incubated at room temperature for 3 and 5 hours. A bacterial culture lacking nanoconjugate was used as a control. The cultures were then centrifuged for 10 minutes at 5000 rpm, and the absorbance of supernatants was measured at 260 nm for estimation of nuclei acids and 280 nm for proteins.

The leakage of proteins through the membrane was further evaluated using a Bradford assay. Briefly, bacterial strains of *E. coli* and *S. epidermidis* were cultured in MH broth. The cells were adjusted to 10^6^ cells per mL and 10 mL of the culture was taken. 100 μL of N-CS-IONP nanoconjugates of ratios (1 : 2) and (1 : 4) were added to the bacterial culture and were kept in an incubator at 37 °C with 150 rpm shaking. Control experiments were carried out without the addition of a nanoconjugate. 1 mL of the sample was kept aside from the culture without the addition of a nanoconjugate. After completion of 2.5, 3.5, and 5.5 hours, the sample was centrifuged at 9000 rpm and the supernatant obtained was immediately frozen at −20 °C. The concentration of proteins was estimated using the Bradford assay.

#### 2.5.4. Evaluation of antibiofilm activity

The impact of the formulated nanoconjugate on biofilm formation was probed by microtiter plate assay. Bacterial cultures of *S. epidermidis* and *Bacillus subtilis* were cultured in nutrient broth and incubated overnight at 37 °C. The resulting bacterial samples were serially diluted to a concentration of 10^6^ cells per mL with LB broth supplemented with 1% glucose. Next, 100 μL of the resulting bacterial culture was added to each well of a 96-well plate for further analysis. IONP and nisin were taken as the negative controls. Moreover, reaction mixtures without treatment were considered positive controls. Following the incubation of the microtiter plate in the incubator for 18 hours at 37 °C, the wells were washed with phosphate buffer (pH – 7.4), and each well was treated with 200 μL of 0.1% crystal violet solution. After incubating the plate for 1 hour, the crystal violet solution was removed. The wells were rinsed twice with phosphate buffered saline for 10 minutes each to eliminate any unbound cells. Thereafter, 200 μL of 95% ethanol was added to each well to dissolve the biofilm, and the plates were dried. Finally, the OD of each well was observed at 600 nm using a Bio-Rad iMark plate reader.

#### 2.5.5. Cytotoxicity assay

The cytotoxicity of IONPs and CS-IONPs was evaluated against a human embryonic kidney (HEK 293, NCCS Pune, India) cell line using an alamarBlue dye reduction assay, as described by Arakha *et al.*^15^ In a 96-well plate, 5000 HEK 293 cells were seeded per well with a suitable medium (DMEM, HiMedia, India), supplemented with 10% foetal bovine serum (FBS). After 24 hours in a CO_2_ incubator, the culture medium was replaced with fresh DMEM medium with varying concentrations of IONPs and CS-IONPs: 2.5, 5, 10, 25, and 50 μg mL^−1^. The IONP and CS-IONP solutions were prepared by dispersing NPs in phosphate buffer (pH 7.4) and sonicated for 20 minutes before use. The cells were then cultured in a CO_2_ incubator for another 24 hours. Before collecting the samples, alamarBlue dye (10% v/v, Invitrogen, USA) was applied to each well and incubated for 2 hours in a CO_2_ incubator before determining the fluorescence intensity at 590 nm with an excitation at 544 nm. The ratio of fluorescence intensity recorded in treated to untreated cells was used to quantify the percentage of viable cells in the untreated (control) and IONPs-treated samples.

## 3. Results

### 3.1. Characterization of synthesized IONPs and CS-IONPs

#### 3.1.1. X-ray diffraction analysis of IONPs and CS-IONPs

**Fig. 1:**
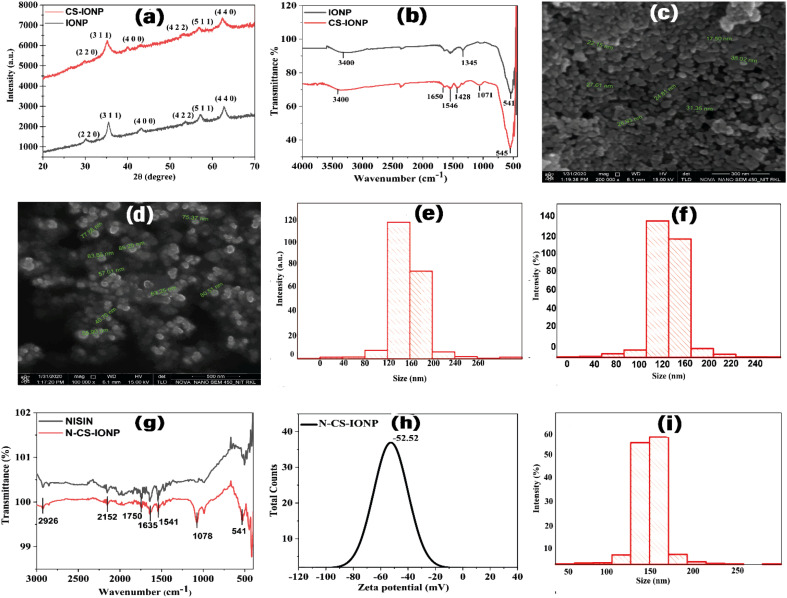
Characterization of IONP, CS-IONP and N-CS-IONP nanoconjugates. (a) Diffraction peaks from XRD analysis of IONPs and CS-IONPs. (b) Bond-level characterization of IONPs and CS-IONPs using FTIR. Morphological analysis of (c) IONPs and (d) CS-IONPs using FE-SEM. DLS analysis of (e) IONPs and (f) CS-IONPs. (g) FTIR analysis of nisin and N-CS-IONP nanoconjugates. (h) Zeta potential analysis of N-CS-IONPs and (i) DLS analysis of N-CS-IONPs.

The diffraction peaks occurring due to X-ray diffraction signals from the set of crystal lattice planes at (220), (311), (400), (422), (511), and (440) corresponding to the 2*θ* values of 30.09°, 35.46°, 43.19°, 53.55°, 57.11°, and 62.76° confirm the formation of Fe_3_O_4_. Similarly, 2*θ* values for CS-IONPs at 29.78°, 35.20°, 40.05°, 52.99°, 56.89°, and 62.20° also correspond to Fe_3_O_4._ The above data confirms the formation of the magnetite inverse cubic spinel phase of both IONPs and CS-IONPs. There is negligible change in the peak positions of both nanoparticles (Fig. 1a), which indicates that the coating of chitosan did not induce any changes in the phases of the nanoparticles. These calculated plane values and peak positions are in good agreement with the standard XRD pattern of Fe_3_O_4_ (JCPDS 00-003-0863). The average crystal sizes of both nanoparticles were calculated to be 14.6 and 14.5 for IONPs and CS-IONPs, respectively, using the Scherrer equation: *D* = (*kλ*/*β* cos *θ*), where *D* is the mean crystal size, *K* is Scherrer's constant (0.89), *λ* is the wavelength of X-rays used for performing the XRD, *β* is the full width at half maximum (FWHM), and *θ* is the Bragg diffraction angle in degrees. Due to its well-resolved state and the absence of any sort of interference, the peak at the lattice plane of (311) was used for the calculation of the average sizes of IONPs, and CS-IONPs. We observed that there was a slight broadening of the peaks and little reduction in peak intensity of CS-IONPs, compared to IONPs, which is mainly accounted for by the chitosan coating.

#### 3.1.2. Bond-level characterizations of IONPs and CS-IONPs

The vibrational frequencies of various bonds present in IONPs and CS-IONPs were analysed using Fourier transform infrared (FTIR) spectroscopy (Fig. 1b). It is noteworthy that the observed peaks at 541 cm^−1^ and 545 cm^−1^ for IONPs and CS-IONPs, respectively, correspond to magnetite, resulting from the Fe–O stretching vibration of tetrahedral sites in the spinel structure present in the compound.^16,17^ Upon coating with chitosan, the peak shifted from 541 cm^−1^ (of IONP) to 545 cm^−1^ (of CS-IONP). This could be due to the hydrogen atoms of the amino groups on the chitosan chain forming hydrogen bonds with the oxygen atoms of the IONP surfaces.^18^ The literature suggested that the likelihood of hydrogen bond formation between the hydrogen and oxygen atoms was increased, which could be attributed to the high reactivity of the oxygen atoms and their propensity to interact with other atoms.^18^ Consequently, it is possible that interactions occurred between the iron atoms on the surface of the IONPs and the nitrogen or oxygen atoms of the functional groups in chitosan.^18,19^ This confirms the presence of IONPs. Compared with the IONP spectrum, the characteristic peaks of chitosan are at 1071 cm^−1^ and 1546 cm^−1^ and arise due the C–N stretching of aliphatic amine and glycosidic bond stretching vibrations^19^ and the NH_3_ group present in the compound, respectively.^19^ These characteristic peaks of chitosan in CS-IONPs demonstrate successful coating. Additionally, the peak observed at 1428 cm^−1^ corresponds to C–N stretching vibrations.^17^ The peaks observed at 1650 cm^−1^ in the IR spectrum of CS-IONPs represent the N–H bending vibration.^17^ The peak at 3400 cm^−1^ indicates the presence of a large number of hydroxyl groups of OH stretching vibrations.^17^ The above data confirm the successful coating of chitosan onto the surface of the IONPs.

#### 3.1.3. Morphological analysis of IONPs and CS-IONPs

The morphological structures of IONPs (Fig. 1c) and CS-IONPs (Fig. 1d) were analysed using FE-SEM. As shown in Fig. 1c and d, both the synthesized IONPs were observed to be almost spherical in shape and have a size range of 30–40 nm, which is consistent with the estimated particle size determined by XRD analysis. Analysis of FE-SEM data indicated that the size of the particles increased slightly upon coating with chitosan.

#### 3.1.4. Hydrodynamic size analysis of IONPs and CS-IONPs

In DLS, the fluctuations in the scattering intensity as a function of time at a fixed scattering vector, q, are measured. The size of the particle is obtained from the Stokes–Einstein equation, 
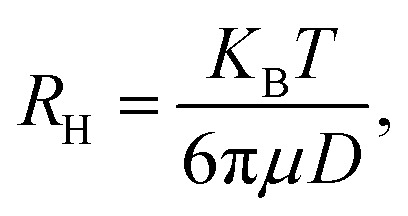
 where *R*_H_ is the hydrodynamic radius, *K*_B_ is the Boltzmann constant (1.38 × 10^−23^ kg m^2^ s^−2^ K^−1^), *T* is the temperature, *μ* is the viscosity, and *D* (the diffusion coefficient) is given by: *D* = *b*/**q**^2^. The values of *λ* and *θ* utilized for the experiment were 632.8 nm and 90°, respectively. The hydrodynamic sizes of the IONPs (Fig. 1e) and CS-IONPs (Fig. 1f) are 137.03 nm and 156.78 nm, respectively. The increase in the hydrodynamic size of CS-IONPs from IONPs suggests the attachment of the positive charge of chitosan over the negatively charged IONP nanoparticle. The attachment of chitosan changes the surface charge density and water molecule arrangement around the nanoparticle, thereby increasing the effective hydrodynamic size of CS-IONPs.

### 3.2. Characterization of nisin-conjugated CS-IONPs (N-CS-IONPs)

#### 3.2.1. Bond-level characterization of N-CS-IONPs

Initially, the conjugation of nisin with CS-IONPs was confirmed by FTIR spectroscopy. According to previous reports, the incorporation of peptide nisin onto the matrix of the biomolecule is solely determined from amide I (stretching vibration, C=O bond) and amide II (bending vibration of the N–H bonds) vibrations of the peptide.^20^ In the case of a nanoconjugate, the peak observed at 1635 cm^−1^ in the IR spectrum corresponds to the amide I band vibrations due to the unordered beta-turn of pure nisin (Fig. 1g).^21–23^ The peak observed at 1541 cm^−1^ in the IR spectrum represents the bending of primary amine.^22^ The peak obtained at 2926 cm^−1^ is attributed to C–H stretching. Similarly, another peak at 1078 cm^−1^ is due to the secondary hydroxyl group.^24^ This implies the conjugation of nisin onto chitosan-coated IONPs (Fig. 1g).^22^ Additionally, the findings suggested that nisin is successfully conjugated upon CS-IONPs, without any conformational changes in nisin.

#### 3.2.2. Surface potential and hydrodynamic size analysis

The surface modification could also be identified by analysing the zeta potential values. For instance, IONPs formed upon reduction by sodium hydroxide have a high negative zeta potential value of −33 mV (Fig. S1a, ESI†). However, chitosan being a cationic polymer, apart from stabilizing the overall system, imparts unique properties such as antibacterial properties as well as neutralizing some of the negative charges present in the CS-IONP conjugate (−44.23 mV) (Fig. S1b†). Literature reports indicate that the stability of nisin-loaded IONPs can be attributed to the electrostatic repulsion that occurs between the carboxylic acid groups and hydroxyl groups when they are in an aqueous solution.^12^ Nisin has the ability to electrostatically bind to acid-stabilized IONPs that have a negative charge. However, the thickness of the peptide corona is minimal and there is only a negligible change in the zeta potential values.^12^ Loading higher concentrations of nisin induces the agglomeration of nanoparticles.^12^ Hence, we have optimized the concentration of nisin and CS-IONPs to formulate nanoconjugates in our experiment. Nisin is electrostatically attached onto the chitosan-coated IONPs. The nanoconjugates were very stable, since the indicated zeta potential value is −52.52 mV (Fig. 1h), which is large enough to prevent particle aggregation. Initially, the hydrodynamic size of the IONPs was found to be 137.03 nm (Fig. 1e), which was further increased to 179 nm for N-CS-IONP nanoconjugates (Fig. 1i). This is due to the attachment of highly negatively charged nisin over the CS-IONPs, thereby slightly increasing the overall effective size.

### 3.3. Effect of N-CS-IONP nanocomposite against Gram-positive and Gram-negative bacteria

#### 3.3.1. Growth kinetics analysis

**Fig. 2:**
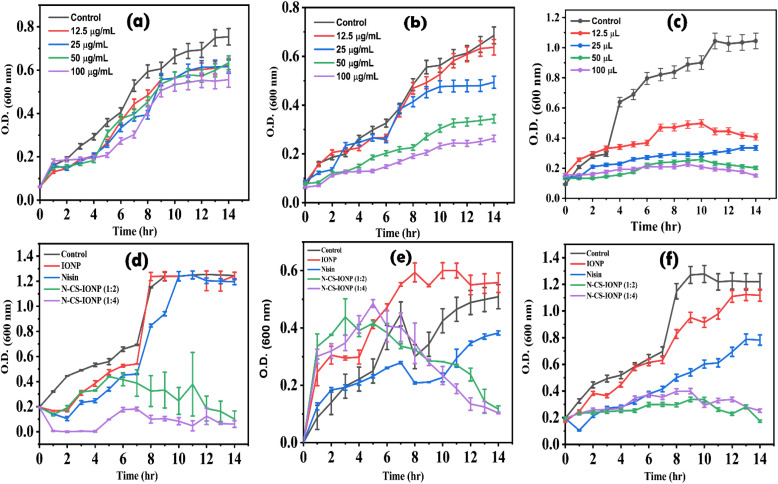
Concentration-dependent growth kinetics analysis of *S. epidermidis* in the presence of (a) IONPs, (b) CS-IONPs and (c) N-CS-IONPs. Comparative growth kinetics analysis of (d) *E. coli*, (e) *S. epidermidis* and (f) *S. aureus* in the presence of IONPs, nisin, and N-CS-IONPs.

Initially, the concentration-dependent antimicrobial activity of synthesized IONPs, CS-IONPs and N-CS-IONPs was evaluated against *S. epidermidis*. As shown in Fig. 2a, insignificant growth inhibition was observed for the studied concentration of IONPs in comparison to the control. However, in the presence of different concentrations of CS-IONP, significant growth inhibition was observed in comparison to the control (Fig. 2b). The CS-IONP nanoparticles showed a uniform decrease in growth of bacteria with an increase in concentration (Fig. 2b). The highest concentration (100 μg mL^−1^) showed a significant decrease in the bacterial population (Fig. 2b). Additionally, the N-CS-IONP nanoconjugate also showed a significant decrease in growth inhibition at lower concentration, such as 25 μL (Fig. 2c). In comparison to IONPs and CS-IONPs, the nanoconjugate showed superior antibacterial ability at very low concentration. Following the concentration-dependent study, growth kinetics studies of IONP, CS-IONP and N-CS-IONP nanoconjugates were then evaluated against both Gram-positive (*S*. *epidermidis* and *S*. *aureus*) and Gram-negative (*E*. *coli*) bacteria. As shown in Fig. 2d–f, IONP activity against both Gram-positive and Gram-negative strains is insignificant. Previous studies have shown that smaller IONPs (less than 20 nm) are generally more effective at inducing bacterial cell damage and reducing bacterial growth than larger particles.^25,26^ Since the size distribution of the above synthesized IONPs *via* co-precipitation is generally larger than 20 nm, rapid agglomeration is the chief cause of the poor antimicrobial activity. Similarly, nisin efficacy is dependent on various environmental factors, such as temperature, pH and the outer membrane of the bacteria.^1^ The microorganisms chosen for this experiment are opportunistic pathogens thriving at an optimum temperature of 36 °C. Hence, nisin activity is limited. However, in the presence of N-CS-IONP (1 : 2) and (1 : 4), significant inhibition was observed against both Gram-negative bacteria such as *E*. *coli*, and Gram-positive bacteria such as *S. epidermidis* and *S*. *aureus* (Fig. 2d–f, respectively). The bacteria were in the mid log phase, *i.e.*, the phase of optimum growth of bacteria, and the addition of the nanoconjugate does not stop the bacterial growth instantly. The growth kinetics of the studied bacteria were observed to re-enter the lag phase for a few hours. The bacterial populations which were not exposed to nanoconjugates or which were tolerant resumed their growth as soon as the dormant phase was over. The data overall indicates that the nanoconjugate is efficient against both Gram-negative and Gram-positive bacteria.

#### 3.3.2. ROS detection

**Fig. 3:**
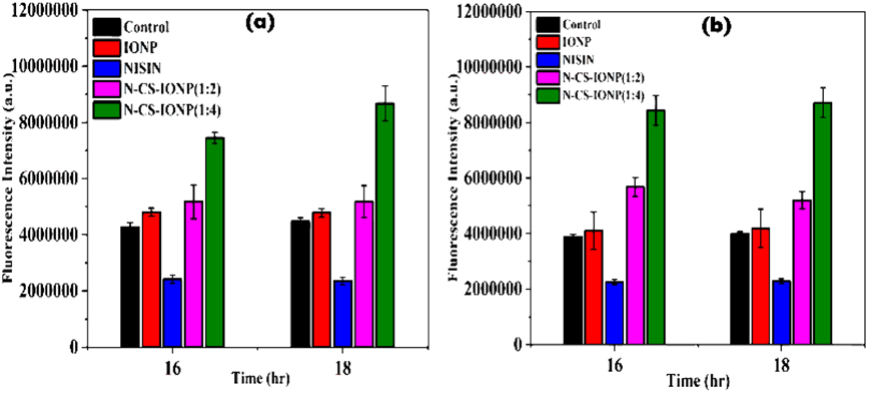
Detection of ROS generated upon interaction of nanocomposite with bacteria: (a) *S. epidermidis* and (b) *E. coli*.

To evaluate the underlying mechanism behind the antibacterial activity of the N-CS-IONP nanoconjugate, the oxidative stress levels were studied using the ROS-specific dye, 2′,7′-dichlorodihydrofluorescein diacetate (DCFH-DA). Reactive oxygen species (ROS) are usually highly reactive free radicals existing in the form of small molecules or ions such as hydrogen peroxide (H_2_O_2_), hydroxyl free radicals (OH˙), or molecular oxygen ions (O_2_˙).^27^ The quantum yield of (DCFH-DA) dye at 523 nm increases significantly in the presence of ROS, indicating their presence.^28^ Therefore, the increase in fluorescence intensity observed at 523 nm over time suggests the production of ROS during bacterial growth at different time intervals, such as 16 hours and 18 hours of growth (Fig. 3a and b). The data presented in Fig. 3a and b shows that nisin and IONPs were not effective in enhancing the production of ROS in bacterial cells. This supports the previous finding that a concentration of 340 nM nisin does not have ROS-mediated antibacterial activity.^7^ In contrast, the nanoconjugates were found to enhance ROS production in both *S. epidermidis* and *V. cholera* bacterial cultures by a 2–3-fold increase in fluorescence intensity.

#### 3.3.3. Leakage of cytoplasmic contents

**Fig. 4:**
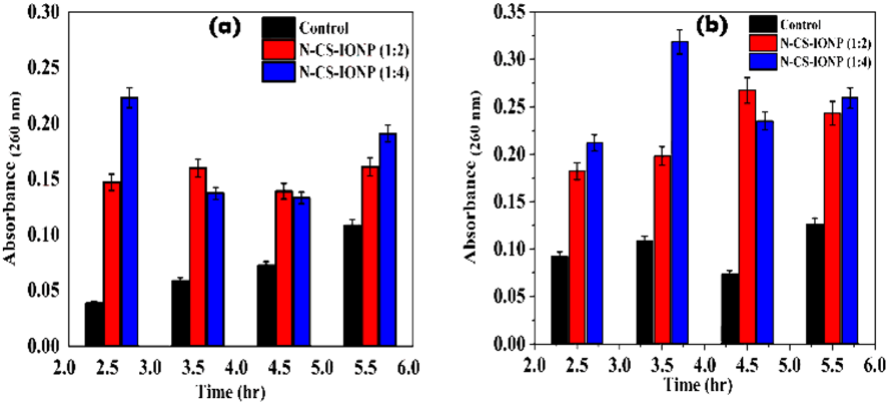
Leakage of nucleic acid measured *via* absorbance at 260 nm for (a) *S. epidermidis* and (b) *E. coli*.

Nanoparticle interaction damages the cellular integrity of the microorganisms. The absorbance by a bacterial culture at 260 and 280 nm upon treatment with nanoconjugates was used to estimate the release of nucleic acids and proteins into the suspensions. We evaluated the amount of nucleic acid leakage at different time intervals upon treatment with nanoconjugates. However, the maximum release of nucleic acid for *S. epidermidis* and *E. coli* was observed after 2.5 and 3.5 hours of treatment with N-CS-IONPs, respectively (Fig. 4a and b).

**Fig. 5:**
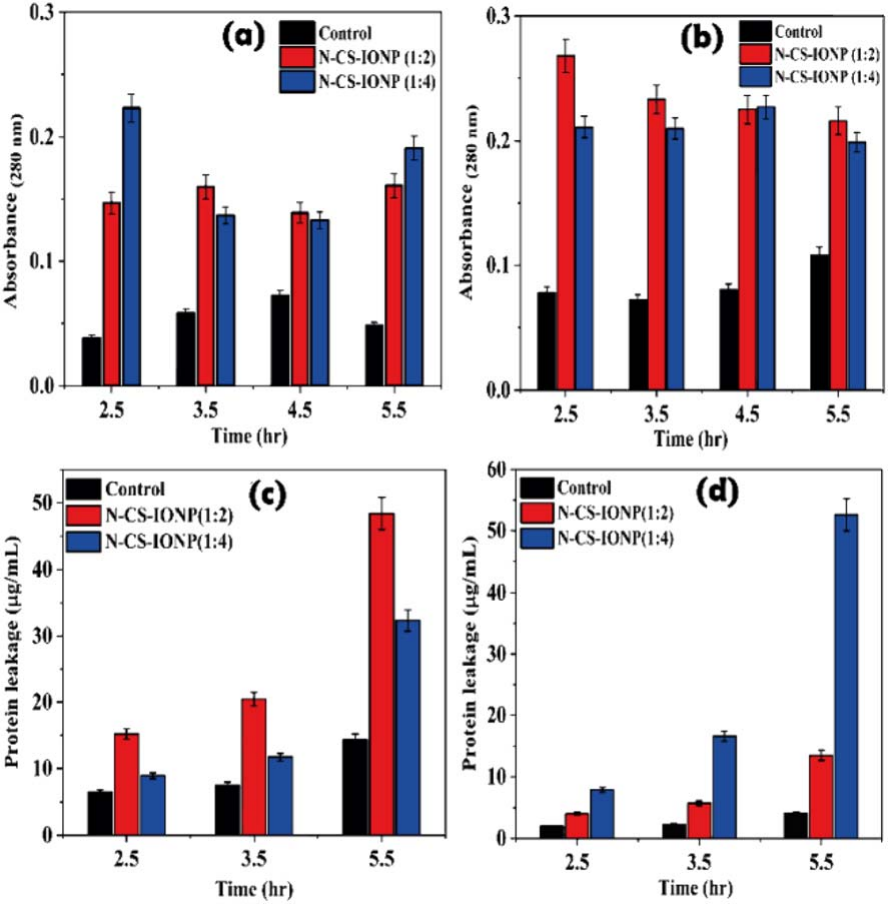
Analysis of protein leakage upon treatment with nanoconjugates. Qualitative analysis of protein leakage by monitoring absorbance for *S. epidermidis* (a) and *E. coli* (b), respectively. Quantitative analysis of protein leakage by a Bradford assay for *S. epidermidis* (c) and *E. coli* (d).

Additionally, we also qualitatively monitored the amount of protein leakage by monitoring the absorbance of bacterial culture at 280 nm upon treatment with nanoconjugates. As shown in Fig. 5a and b, we observed a significant amount of protein leakage at different time intervals for both ratios of nanoconjugates for the studied bacteria *S. epidermidis* and *E. coli*, respectively.

The amount of leakage of protein was further quantified using a Bradford assay. To gain knowledge about membrane damage, protein leakage estimation was conducted by evaluating the amount of protein leakage by the Bradford assay method. The Bradford method is a protein quantification technique based on the change in absorbance of Coomassie brilliant blue G-250 dye in an acidic solution.^29^ The dye binds to proteins through electrostatic interaction to the sulfonic groups, primarily to arginine residues, but also to a lesser extent to histidine, lysine, tyrosine, tryptophan, and phenylalanine.^29^ The binding of the dye to protein occurs rapidly. The resulting protein–dye complex has a stable blue colour that persists for up to 1 hour, providing sufficient time to measure a large number of samples accurately.^29^ The bacterial proteins extracted were quantified against BSA standards. Bradford assays of BSA as a standard resulted in a highly reproducible response pattern, with a statistical analysis giving a standard deviation of around 1% of the mean value for the assay.^30^ It can be deduced from the protein leakage data that N-CS-IONPs are more effective against both Gram-positive bacteria (*S. epidermidis*) (Fig. 5c) and Gram-negative bacteria (*E. coli*) (Fig. 5d). However, the amount of protein leakage for *S. epidermidis* and *E. coli* was found to be 48.42 μg mL^−1^ and 52.62 μg mL^−1^, respectively.

#### 3.3.4. Antibiofilm activity of N-CS-IONP nanoconjugate

**Fig. 6:**
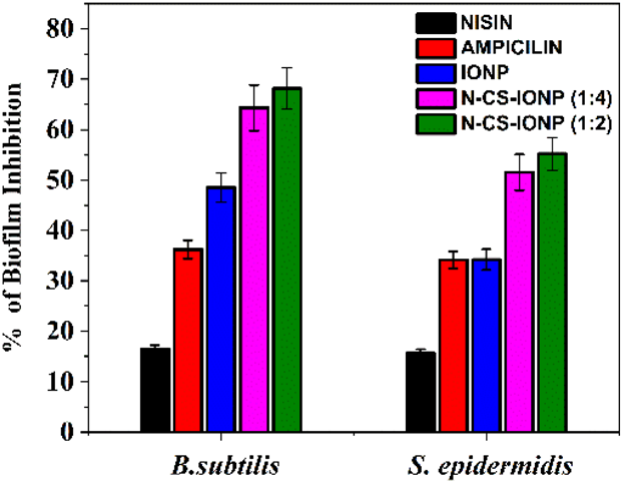
Antibiofilm study of the nanocomposite against *B. subtilis* and *S. epidermidis*.

Nisin has been profiled as one of the most effective AMPs in the prevention of biofilm formation. IONPs have recently been proven to be a highly efficacious tool for the site-specific treatment of biofilms formed by antibiotic-resistant *S*. *aureus*.^31^ As observed from our studies (Fig. 6), upon treatment with nanoconjugate, biofilm formation was decreased significantly in the case of both *B. subtilis* and *S. epidermidis*. Ampicillin, a broad-spectrum antibiotic, was taken as a positive control and nisin and IONP served as negative controls. The concentration of nanoconjugate showed a greater percentage of inhibition compared to the controls, confirming that the conjugation of nisin on CS-IONP synergizes their individual efficacy.

#### 3.3.5. Cytotoxicity assay

**Fig. 7:**
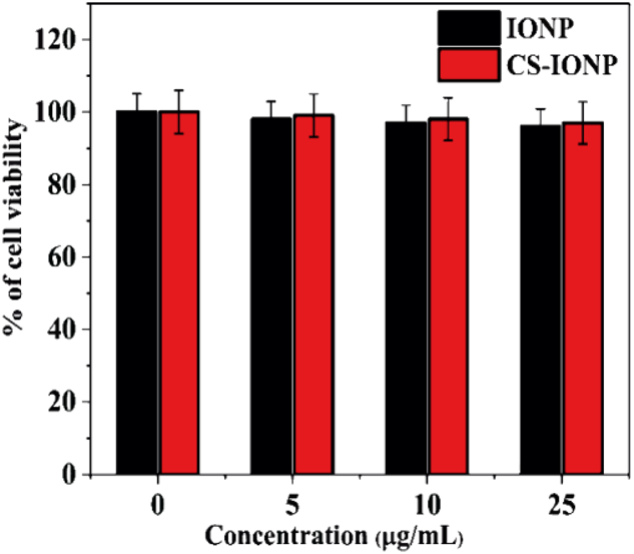
Cytotoxicity study of IONPs and CS-IONPs against the human embryonic kidney (HEK 293) cell line.

To gather key information about cell death and cell survival in the presence of nanoparticles, a cell viability assay was performed. The data in Fig. 7 indicates the relationship between different concentrations of IONPs and CS-IONPs and viability of HEK cells. A slight decrease in cell viability is noticed with an increase in the studied concentration of IONPs with respect to the control (0 μg mL^−1^). As previously reported, the cytotoxicity of IONPs arises due to the high dissolution of Fe ions.^32^ It is apparent that to increase the cytocompatibility of the nanoconjugate, the core *i.e.* IONP is coated with oligosaccharide chitosan. The results from the cytotoxicity assay indicate that the coating generously accentuated the biocompatibility of IONPs for *in vivo* biomedical applications.

## 4. Discussion

Oxides of iron (Fe_3_O_4_) *via* a co-precipitation method were obtained by aging a stoichiometric mixture of ferric and ferrous salts in aqueous media according to the following chemical equations:^4^

2 Fe_3_O_4_ formation: Fe^2+^ + 2Fe^3+^ + 8OH^−^ → Fe_3_O_4_ + 4H_2_O

3 γ-Fe_2_O_3_ formation: Fe_3_O_4_ + 2H^+^ → γ-Fe_2_O_3_ + Fe^2+^ + H_2_O

The thermodynamics of reaction (2) predict the complete precipitation of Fe_3_O_4_ at a pH in the range of 8 to 14, by keeping the stoichiometric ratio constant at 2 : 1 (Fe^3+^ : Fe^2+^) and maintaining an oxygen-free environment. The pH of the suspension, the level of acidity and the ionic strength of the reaction medium highly regulate magnetite size formation by affecting the overall chemical composition of the surface of the crystal and the electrostatic charge on the surface of the nanoparticles.^33^ The formed magnetite (Fe_3_O_4_) is inadequately stable, and it gets oxidized and converted to maghemite (γ-Fe_2_O_3_) according to reaction (3) due to the presence of oxygen. This happens due to the presence of iron atoms at the surface of iron oxide, which function as Lewis acids and form coordination bonds with molecules possessing lone-pair electrons. On account of this, for the synthesis of IONPs, the reaction was carried out at pH 11 by the addition of NaOH. Being benign to humans because of their acidic dissolution property, the release of IONPs will be negligible when injected *in vivo*.^10^ Therefore, a high dilution of IONPs is generally used for *in vivo* and *in vitro* applications of IONPs.^10^ Once inside the biological milieu, reversible dispersant adsorption occurs, which leads to agglomeration of iron oxide.^10^ The effects of NP agglomeration on these applications can be severe. Because NP size cannot be directly measured once injected into a living body, agglomeration of NPs inside a biological milieu is difficult to assess.^10^ This results in a poorly defined system in which examination of the effectiveness or targeting of NPs is not specific. Thus, to provide a decisive advantage for biomedical applications, chitosan is utilized as a stabilizing agent regulating the electrostatic and steric repulsion forces, to achieve a decent stabilized magnetite, thereby preventing further oxidation. Chitosan being a cationic hydrophilic polymer is a biocompatible molecule, inert against air, with its amino group exposed to interact with the hydroxide functional group present at the IONP surface. Hence, chitosan maintains the stability of synthesized IONPs in aqueous solutions such as water by preventing the coordinated iron atoms from readily dissociating. IONPs were mixed with chitosan at a ratio of 5 : 2. FTIR, zeta and DLS analysis confirmed the successful coating of chitosan onto the IONPs. In addition to providing stability, chitosan possesses other desirable properties, such as biocompatibility, biodegradability, and bactericidal activity. The literature has well documented the conjugation of IONPs with chitosan, which readily enhanced the bactericidal action of IONPs against various bacteria like *B. subtilis* and *E. coli* due to ROS generation.^34^ In this context, a separate study evaluated the antibacterial efficiency of Fe_3_O_4_@SiO_2_ MNPs and chitosan-coated Fe_3_O_4_@SiO_2_@CS composites against vancomycin-intermediate (VISA strain) *S. aureus*. The results of the study indicated that the addition of chitosan to the magnetic nanoparticles (MNPs) increased the sensitivity of the VISA strain (*S. aureus*), with chitosan-coated MNPs exhibiting up to a 2-fold reduction in both the minimum inhibitory concentration (MIC) and the minimum bactericidal concentration (MBC) compared to free MNPs.^13^ Previous research demonstrated the antimicrobial activity of chitosan, which specifically disrupts the microbial membrane while displaying no toxicity towards mammalian somatic or tumoral cells.^35^ In a separate investigation, chitosan polymer exhibited high uptake by mammalian cells and specific binding to the negatively charged bacterial membrane, allowing for the targeted release of tetracycline at the desired site. These cationic chitosan-coated gold nanoparticles were able to significantly reduce the survival rate of intracellular *S. aureus* from 15% to 2.5% compared to treatment with tetracycline alone.^2^ Nanoparticle stabilization is currently under study to revive the compromised activity and impaired stability of AMPs against microorganisms due to proteolytic enzymes, temperature and buffers.^2^ Along the above stated lines, it is reported that physically immobilising peptides on the NP surface could improve the stability and lifespan of AMPs.^2^ These peptide–NP complexes have longer bactericidal action against *Listeria monocytogenes* than free peptides, lasting up to 21 hours, because of greater electrostatic and hydrophobic contacts between the NPs and peptides. Using a different technique, Gupta *et al.* have mentioned that covalently linked cationic peptide cecropin–melittin with AuNPs, resulted in improved antibacterial action even in the presence of large concentrations of proteolytic enzymes.^2^ The synergy arising between nanoparticles, stabilizing agent and the antimicrobial peptide provides an enhanced avenue to combat multidrug-resistant microorganisms.^2^ Therefore, in our study, we initially functionalized IONPs with chitosan and formulated a novel nanoconjugate by allowing an AMP like nisin to be adsorbed onto the surface of CS-IONPs. Nisin consists of a linear peptide chain of 34 amino acids that are modified by the addition of five thioether rings, conferring on it unique properties, including its ability to interact with bacterial membranes.^36,37^ The classical pathway of nisin is primarily by interaction with the bacterial membrane by binding to the lipid II molecule through its C-terminal domain, which contains a conserved motif called the lipid II-binding motif (LIM).^38^ This binding disrupts the synthesis of the peptidoglycan layer by the formation of pores due to the amphipathic nature of nisin, which allows its insertion into the bacterial membrane, subsequently leading to cell death.^38^ The formation of pores also leads to the leakage of intracellular contents, leading to cell death. Here, we have prepared nanoconjugates and labelled them N-CS-IONP (1 : 2) and N-CS-IONP (1 : 4), where the nisin concentration was kept constant at 1 mg mL^−1^, while the CS-IONP concentration was varied at 2 mg mL^−1^ and 4 mg mL^−1^. The peptide nisin is electrostatically attached onto the positively charged chitosan, as evident by the zeta analysis. The conjugation with CS-IONPs does not confer any significant change to the structure of the peptide, as evident from the FTIR data.

The literature even reports numerous studies on the antimicrobial activity of nisin upon conjugation with nanoparticles. In this context, Behzadi *et al.* demonstrated that mesoporous silica (MSN)-loaded nisin NPs exhibited antimicrobial activity against *S. epidermidis* under various conditions, with negligible cytotoxic effects on L929 cells (mouse fibroblast).^39^ Additionally, Pandit *et al.* developed a nanosilver–nisin bioconjugate which was incorporated into agar film for treatment against specific food spoilage germs. The agar film established maximum activity against *Pseudomonas fluorescens* and minimum activity against *Fusarium moniliforme*.^40^ Numerous studies therefore provide sufficient insight into the heightened activity of nisin upon conjugation with nanomaterials, such as lipids, polysaccharides^41^ and metals.^42,43^

This nanoconjugate can be interpreted as an antimicrobial peptide (AMP) carrier model since the nanoconjugate particles have a molecular size that is large enough to hinder removal *via* the efflux pump of Gram-negative bacteria, yet small enough to traverse the pore formed by nisin. According to our findings, 50 μL of N-CS-IONP nanocomposite exhibits notable bactericidal and bacteriostatic effects towards both Gram-positive and Gram-negative strains. For Gram-positive strains, the antibacterial effects can be stressed upon the release of ROS which arise from the attachment of nanoconjugate to the bacterial cell surface.^13^ It can also be hypothesized that attachment of the nanocomposite also releases nisin, which forms pores so the generated Fe^3+^ ions are transported inside the cells and interact with biomolecules such as proteins and nucleic acids, impairing enzymatic activity, altering protein structures, and interrupting DNA strands, ultimately leading to cell death.^13^ This is evident from the cytoplasmic leakage assay. The nanoconjugate showed maximum leakage of cytoplasmic content at 2.5 hour. In contrast, Gram-negative bacteria have a much thinner peptidoglycan layer and an outer membrane composed of lipopolysaccharides (LPS).^38^ LPS acts as a barrier that can prevent nisin from reaching the peptidoglycan layer and binding to lipid II.^38,44^ Additionally, the outer membrane of Gram-negative bacteria contains efflux pumps that can actively pump out nisin, reducing its concentration in the bacterial cell and making it less effective.^44^ For Gram-negative bacteria, crowding of metal nanoparticles on the bacterial surface triggers excessive generation of Fe^3+^ ions and ROS, leading to cell death.^13^ Therefore, cell disruption occurred with time and the highest leakage of cytoplasmic contents was observed at 3.5 hour and also with a higher concentration ratio of nanoconjugate (N-CS-IONP (1 : 4)).

**Fig. 8:**
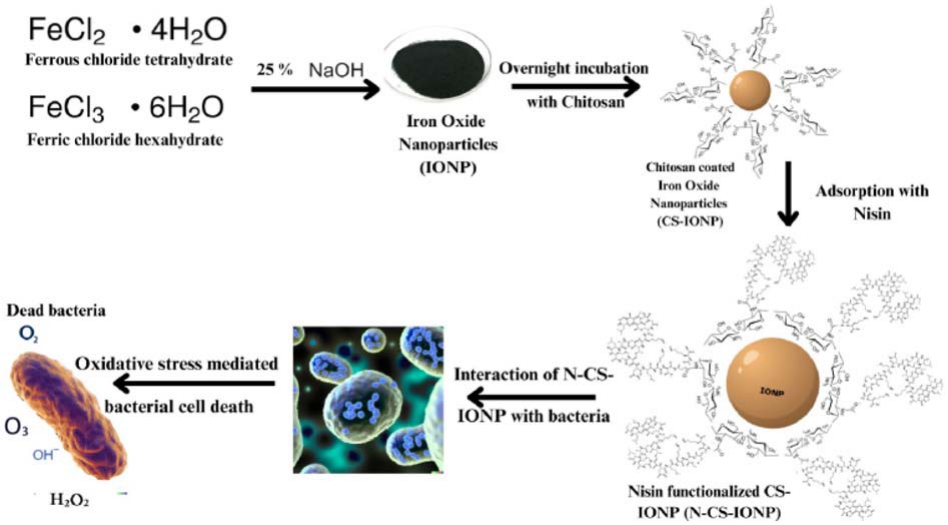
Schematic representation showing the synthesis route of N-C-IONP nanoconjugate and interaction between nisin, chitosan and IONPs. The nanocomposite is evaluated against Gram-positive and Gram-negative microorganisms for determination of its antimicrobial efficacy.

Subsequently, biofilms even play a vital role in antimicrobial resistance (AMR), since the defensive matrix they generate allows microbes to survive against antimicrobial agents. Microorganisms within a biofilm have a higher resistance to antibiotics, making them more resistant than planktonic microbes. Therefore, combating biofilm-associated antimicrobial resistance necessitates formulations capable of targeting the biofilm-embedded bacteria. Treatment with the N-CS-IONP nanoconjugate showed significant decrease in the formation of *B. subtilis* and *S. epidermidis* biofilm. The mechanism behind this inhibition could be attributed to disrupting the cell-to-cell signalling system that regulates biofilm formation and other cooperative behaviours.^45^ Since nanoconjugate treatment leads to a significant decrease in the microbial population, which consequently results in outer membrane damage, thereby leading to impaired quorum sensing mechanisms. The antimicrobial and antibiofilm activity of the nanoconjugate is notably far superior to that of IONPs, CS-IONPs and even free nisin, which also confirms the proposition that loading of drugs can significantly improve their antimicrobial propensities in contrast to simple nanoparticles. Fig. 8 shows a schematic representation elucidating the chemical synthesis of IONPs and adsorption of nisin onto chitosan-functionalized IONPs followed by oxidative stress mediated bacterial cell death.

## 5. Conclusions

This current work exploits the electrostatic assembly approach for the facile conjugation of nisin onto the surface of chitosan-coated magnetite. The chitosan coating ensured the cytocompatibility of the IONPs as well as synergizing the activity of the N-CS-IONP nanoconjugate for eradicating a bacterial population. Moreover, the primary antimicrobial mechanism of the nanoconjugate can be attributed to the pore formation mechanism in the cell membrane, as evident from other studies focusing on nisin.^37^ This is evident from the ROS analysis and cytoplasmic leakage study. A systematic research approach has been adopted, which has highlighted that a minimal dosage of the nanoconjugate is significantly effective against both Gram-positive and Gram-negative bacterial populations. The work even focused on the biofilm degradation ability of the nanocomposite. Future work would focus on the usage of the nanoconjugate against other pathogens as well as more standardized biocompatibility tests to be used in multitudinous applications in the biomedical sector.

## Author contributions

Lipsa Leena Panigrahi: conceptualization, investigation, methodology, validation, writing original draft; Shashank Shekhar: data curation; Banishree Sahoo: data curation; Manoranjan Arakha: conceptualization, formal analysis, methodology, supervision, review & editing.

## Conflicts of interest

The authors have declared that they have no conflict of interest.

## Supplementary Material

RA-013-D3RA04070D-s001
