# Supplementary material for: Adsorption of antimicrobial peptide onto chitosan-coated iron oxide nanoparticles fosters oxidative stress triggering bacterial cell death
Source: RSC Adv. 2023 Aug 25;13(36):25497–507. doi: 10.1039/d3ra04070d (PMC10450573; doi:10.1039/d3ra04070d)
Supplement: RA-013-D3RA04070D-s001 [file RA-013-D3RA04070D-s001.pdf]

Electronic Supplementary Material (ESI) for RSC Advances.
This journal is © The Royal Society of Chemistry 2023

Supplementary information

Adsorption of antimicrobial peptide onto chitosan coated iron oxide nanoparticles fosters oxidative stress triggering bacterial cell death

Lipsa Leena Panigrahi[a], Shashank Shekhar[b], Banishree Sahoo[a], Manoranjan Arakha[a]*

[a.] *Center for Biotechnology, Siksha 'O' Anusandhan (Deemed to be University), Bhubaneswar, 751003, Odisha, India. E-mail: marakha@soa.ac.in*
[b.] *Indian Institute of Technology, Hyderabad, India*

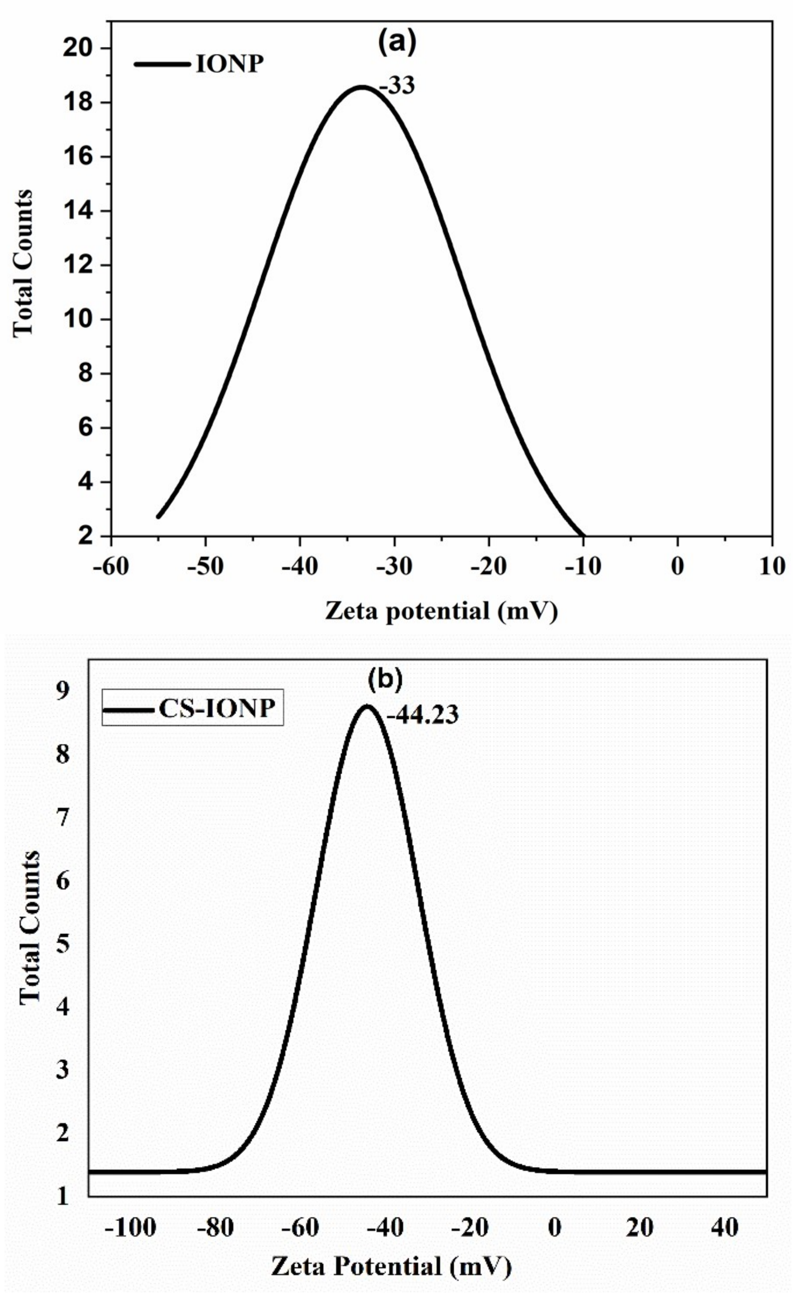

Fig. S 1. Zeta potential analysis of (a) Iron oxide nanoparticles (-33 mV) (b) Chitosan coated Iron oxide nanoparticles (CS-IONP) (-44.23 mV)
